# Are death and loss to follow-up still high in people living with HIV on ART after national scale-up and earlier treatment initiation? A large cohort study in government hospital-based setting, Myanmar: 2013-2016

**DOI:** 10.1371/journal.pone.0204550

**Published:** 2018-09-25

**Authors:** Zaw Zaw Aung, Myo Minn Oo, Jaya Prasad Tripathy, Nang Thu Thu Kyaw, San Hone, Htun Nyunt Oo, Suman S. Majumdar

**Affiliations:** 1 National AIDS Programme, Department of Public Health, Ministry of Health and Sports, Nay Pyi Taw, Myanmar; 2 International Union Against Tuberculosis and Lung Disease, Mandalay, Myanmar; 3 International Union Against Tuberculosis and Lung Disease, South East Asia Regional Office, New Delhi, India; 4 Burnet Institute, Melbourne, Australia; National and Kapodistrian University of Athens, GREECE

## Abstract

**Setting:**

Myanmar National AIDS Program has had significant scale-up of services and changes in CD4 eligibility criterion for ART initiation from 2013 to 2016. This study assessed early death within 6 months and attrition (death and loss to follow-up, LTFU) after ART initiation and their associated factors.

**Design:**

A retrospective cohort study on people living with HIV (PLHIV >15 year of age) enrolled at three specialist hospitals in Yangon from 1^st^ June 2013 to 30^th^ June 2016. Cox regression was used to calculate hazard ratios (HRs) of early death and attrition.

**Results:**

Of 11,727 adults enrolled, 11,186 (95%) were initiated on ART, providing 15,964 person-years of follow-up. At baseline, median age was 36 years [IQR: 30–43], 58% were men and median CD4 count was 151 cells/mm3 (IQR: 54–310). There were 733(6%) early deaths, 961(9%) total deaths and 1371 (12%) LTFU during the study period. Independent risk factors for early death were older age (41–50 and ≥51 years) [aHR 1.38, 1.07–1.78 and 1.68, 1.21–2.34], male (1.84, 1.44–2.35), low weight (2.06, 1.64–2.59), bedridden, (3.81, 2.57–5.66) and CD4 count ≤ 50 cells/mm3 (6.83, 2.52–18.57). In addition to above factors, high attrition was associated with an abacavir-based regimen.

**Conclusion:**

Although there was a low rate of early deaths, patients were being diagnosed late and there was a high attrition rate from specialist hospitals. Concerted effort is required to increase early diagnosis and ART initiation, and strengthen community systems for HIV care to achieve ambitious goal of ending AIDS epidemic by 2030.

## Introduction

The global response to human immunodeficiency virus (HIV) and acquired immunodeficiency syndrome (AIDS) has resulted in 45% reduction in AIDS-related deaths from a peak of 2 million in 2005 to 1.1 million in 2015.[1] This has been largely driven by the scale up of anti-retroviral treatment (ART) coverage. The global strategy of The United Nations Programme on HIV/AIDS (UNAIDS) to end the AIDS epidemic by 2030 aspires to zero new infections, AIDS-related deaths and discrimination and adopts the 90-90-90 targets: 90% of PLHIV know their status, 90% of PLHIV who know their status receive treatment and 90% of PLHIV on treatment have suppressed viral load. In line with this, Myanmar recently released 3^rd^ National Strategic Plan on HIV/AIDS to guide its response to HIV at local, national and regional level.[2]

With an estimated national HIV prevalence at 0.59%, epidemiological modeling in 2014 reported that approximately 210,000 people were living with HIV and 9,000 new infections and 11,000 deaths occurred in Myanmar.[3,4] Moreover, it is estimated that around 130,000 people were receiving ART by the end of December 2016, which accounts for 63% of all eligible PLHIV population in Myanmar.

The National AIDS Programme (NAP) in Myanmar commenced the ART programme at 6 centres in 2005 and scaled up to 113 centres in 2016. The program also started decentralizing ART provision services to the townships in 2012 to reduce the workload at ART centres and increase access to care and services for PLHIV outside of major cities. Currently 140 decentralized ART sites are operational. Two previous studies from public and private sector cohorts of PLHIV on ART in Myanmar show that between 9 and 14% died and up to 7% were lost to follow up over a range of 1–5 years.[5,6] Risk factors associated with mortality and loss-to-follow-up (LTFU) have also been documented in these studies. However, further studies of mortality and unfavorable programmatic outcomes in public ART cohorts in Myanmar have not been conducted in the past 4 years during the period of service scale-up and decentralization by the NAP and changes in CD4 eligibility criterion for ART initiation. In this study, we assessed early mortality, attrition and their associated risk factors among PLHIV (> 15 years of age) enrolled in three large government hospitals (Mingalardon, Waibargi and Tharketa Specialist Hospitals) in Yangon.

## Materials and methods

We conducted a retrospective cohort study on all adult PLHIV (>15 year of age) enrolled at three hospitals specializing for HIV care in Yangon, Myanmar during the period of 1^st^ June 2013 to 30^th^ June 2016.

### Setting

Myanmar is administratively sub-divided into 75 districts, 330 townships, wards, and villages. The NAP central office in Nay Pyi Taw plans and implements its activities through 46 AIDS/STD (sexually transmitted disease) teams at the district level. The NAP has been working in collaboration with international non-government organisations (iNGOs) since 2005 and has been increasing its own capacity to now manage 113 ART centres that can initiate ART through medical doctors and physicians who are trained in HIV treatment and care. All ART centres provide free HIV treatment and care with the support from the Global Fund against AIDS TB and Malaria (GFATM) in additional to the Government’s contribution. The three government hospitals, Mingalardon Specialist Hospital, Waibargi Hospital and Tharketa Specialist Hospital are all located in the city, Yangon. Waibargi Specialist Hospital is the first ART centre in Myanmar (established in 2005) where more than 1,800 adult PLHIV are receiving ART. Mingalardon Specialist Hospital is the largest ART centre and is now providing care for nearly 8,300 PLHIV. Tharketa Specialist Hospital has nearly 4,000 PLHIV on ART.

Voluntary Confidential Counseling and Testing (VCCT) for HIV in Myanmar is available at all ART centers, decentralized ART sites, government hospitals and through non-government organisations (NGOs). Township TB centers have VCCT services for HIV to TB and presumptive TB cases. On diagnosis with HIV, the individual is referred to the ART centre where they are enrolled in the pre-ART register. New diagnoses have their referral source classified as either out-patient, in-patient, TB/HIV, or prevention of mother to child transmission. On the first visit to the clinic, baseline clinical assessment and laboratory investigations including cluster of differentiation (CD)4 count, haemoglobin level and liver function tests are performed. In response to new WHO guidelines, the CD4 count criterion to initiate ART has been increased from ≤ 200 cells/mm^3^ in 2011 to ≤350 in 2012 and again to ≤500 in March 2015.[7,8] The criteria for eligibility for ART regardless of CD4 count are WHO clinical stage 3 or 4, pregnant and breastfeeding women with HIV, co-infection with active TB, hepatitis B co-infection, HIV-positive individual in a serodiscordant couple, and HIV infected key populations such as female sex workers (FSW), men who have sex with men (MSM) and people who inject drug (PWID).[8]

In Myanmar, ART can only be initiated by specialist physicians and medical doctors trained by NAP at new recruitment and hands-on training. The ART regimen changed from a stavudine-based (d4T) to tenofovir-based (TDF) regimen in 2012 and stavudine was phased out in 2015.[8] The current first line regimen recommended by NAP for adults is tenofovir, lamivudine and efavirenz (TDF+3TC+EFV). On ART initiation the individual is then enrolled in the NAP ART register. Patients are scheduled for an initial follow-up visit 1–2 months after ART initiation, and 3 monthly visits thereafter. Clinical assessments are done every visit and laboratory investigations at least every 6 months, depending on the patient’s condition. All services are provided free of cost to the patients. Treatment status or outcomes is also recorded for every visit as retained in care, loss-to-follow-up (LTFU), death or transferred out. Three definitions of programmatic outcomes of PLHIV who were put on ART were 1) “retained in care” defined as not missing scheduled follow-up visit, 2) LTFU, defined as not coming for follow-up visit within 3 months after last scheduled date, 3) death, due to any reasons.

HIV care is decentralized by the NAP to the township and station levels. The criteria to decentralize PLHIV to the township nearest to their place of residence are taking a first-line regimen, being on ART more than 6–12 months and having a stable clinical condition. Peer volunteers also support HIV care through funding from the current Global Fund for AIDS, tuberculosis (GFATM) grant. They are paid an incentive per activity that include enrollment, peer counseling, providing psychosocial support and outreach activities, including tracing those lost to care. The tracing activities are carried out weekly or fortnightly.

At the ART centres, every patient receives a dedicated OPD booklet at registration visit, where the attending doctors enter clinical details. Then the doctors enter more comprehensive data related to the patient using another standardized recording tool called Treatment Card (as known as white card). As soon as the clinic session is over, the data in these records are again electronically captured into a dedicated MS excel datasheet by trained data entry operators and nurses. Data quality control is followed using data validation to restrict invalid values, in order to minimize errors and ensure data consistency.

### Data management and analysis

Data from the study were collected from the electronic ART registers (MS excel) from the three centers and merged into a single dataset. Variables collected for socio-demographic and clinical information included age, sex, weight in kilograms and height in centimeter, marital status, literacy, employment, baseline WHO clinical staging, CD4 count (cells per cubic milliliter, cells/mm^3^), haemoglobin level (grams per deciliter), date of enrollment, date of ART initiation, outcome (death, loss to follow up, in care, transfer out) and date of outcome. In this study we assigned participants two study outcomes: 1) **early death** defined as any death within 6 months after ART initiation and 2) **attrition**, which refers to a composite outcome of all patients who died or were LFTU (>3 months after their last clinic visit) due to any reasons, based on studies where most of LTFU were reported to become unascertained deaths.[9,10] For those who were retained on ART, the dates were censored on the last scheduled date up to 31^st^ December 2016. We defined “retention in care” as being those who retained in care at the end of the study period, excluding any cases of transfer-out.

Socio-demographic and clinical characteristics were described using frequency and proportion for categorical variables and median and interquartile range (IQR) for continuous variables. Incidence rates of early death within 6 months and attrition after ART initiation were estimated and expressed as rate per 1000 person-years (PYs). Unadjusted hazard ratios (HR) were calculated to find the association between the key outcomes and baseline characteristics. Those found statistically significant during bivariate analysis (p<0.2) were entered into a Cox proportional hazards regression model and adjusted HRs were calculated. Model selection was based on comparisons of change in model-2 log-likelihood in backward stepwise manner. Proportionality assumptions were tested using Schoenfield residuals, log-log plots, and observed versus predicted survival plots. Cumulative probabilities of unfavorable outcome after ART initiation across key variables were described using Kaplan-Meier estimates. Statistical significance was set at 0.05 and 95% confidence interval (CI) were estimated for incidence rates and HR of both early death and unfavorable outcomes after ART initiation. All analyses were performed using Stata version 14.0 for Windows (Stata Corp, College Station, Texas, USA).

This study was approved by the Ethics Review Committee of Department of Medical Research, Ministry of Health and Sports, Myanmar and the Union Ethics Advisory Group, Paris, France. Patient identifiers used in this study were de-identified before the data analysis.

## Results

### Socio-demographic and clinical characteristics

Between 1^st^ June 2013 and 30^th^ June 2016, a total of 11,727 adults were enrolled in HIV care in these three study hospitals and their socio-demographic characteristics are displayed in Table 1. Median age at enrollment was 36 years (IQR: 30–43) and more than half of them were male and married. Almost all patients (95%) reported heterosexual contact as their risk factor for HIV transmission, however data were missing for 50%. Of 9,458 patients (81%) with available CD4 count at baseline, the median was 151(IQR: 54–310). At baseline, approximately half of the PLHIV were classified as WHO clinical stage 3 or had a CD4 count ≤200 cells/mm^3^ (58%) and one in four a ≤50 cells/mm^3^. The median weight as 50 kg (IQR: 44–56) and 12% of patients were bedridden. Only 6% had anemia at baseline, however 17% of the cohort was missing haemoglobin values; of these, 62% had WHO clinical stage 3 and 4 disease. Of enrolled patients, 11,186 (95%) were put on ART and tenofovir-based regimens were used in the majority, as per national guidelines.

**Table 1:** Socio-demographic, clinical and immunological characteristics of adult people living with HIV who were enrolled in three hospitals specializing for HIV care in Yangon, Myanmar, 1st June 2013 to 30th June 2016.

Socio-demographic characteristics		n (%)
**Total**		11727 (100)
**Sex**	Female	4929 (42)
** **	Male	6798 (58)
**Age at enrollment (Years)**	≤25	1053 (9)
** Median (IQR) = 36 (30–43)**	26–30	2032 (17)
** **	31–40	4949 (42)
	41–50	2627 (22)
** **	≥51	1054 (9)
** **	Not recorded	12 (0)
**Baseline Weight in Kg**	<44 Kg	2301 (24)
** Median (IQR) = 50 (44–56)**	44–56 Kg	4958 (52)
** **	>56 Kg	2293 (24)
** **	Not recorded	2175 (19)
**Year of ART initiation**	2013	1338 (12)
** **	2014	3692 (33)
** **	2015	4357 (39)
** **	2016	1799 (16)
** **	Not recorded	541 (5)
**Marital status**	Single	2432 (21)
** **	Married	6619 (57)
** **	Separated	757 (6)
** **	Divorced	877 (7)
** **	Widowed	1014 (9)
** **	Not recorded	28 (0)
**Literacy status**	Illiterate	1281 (11)
** **	Literate	10399 (89)
** **	Not recorded	47 (0)
**Employment status**	Employed	8537 (73)
** **	Unemployed	3133 (27)
** **	Not recorded	57 (0)
**Alcohol consumption**	Habitual	799 (7)
** **	Social	2779 (24)
** **	Never	8035 (69)
** **	Not recorded	114 (1)
**Transmission risk for HIV**	MSM	50 (1)
** **	SW	9 (0)
** **	Heterosexual	5588 (95)
** **	PWID	54 (1)
** **	Mother to child transfusion	15 (0)
** **	Blood transfusion	66 (1)
** **	Unknown	87 (1)
** **	Not recorded	5858 (50)
**History of previous ART**	No	10441 (90)
** **	Yes	1164 (10)
** **	Not recorded	122 (1)
**Baseline Performance**	Normal activity	9991 (87)
** **	Bedridden less than 50%	262 (2)
** **	Bedridden for more than 50%	1200 (10)
** **	Not recorded	274 (2)
**WHO clinical stage**	Stage 1	3281 (29)
** **	Stage 2	1749 (15)
** **	Stage 3	3864 (34)
** **	Stage 4	2521 (22)
** **	Not recorded	312 (3)
**Baseline Hemoglobin level**	<7 g/dl	631 (6)
** Median (IQR) = 11 (9.2–12.5)**	7–11 g/dl	5850 (60)
** **	>11 g/dl	3254 (33)
** **	Not recorded	1992 (17)
**Baseline CD4 counts**	≤50 cells/mm3	2262 (24)
** Median (IQR) = 151 (54–310)**	51–200 cells/mm3	3226 (34)
** **	201–350 cells/mm3	2057 (22)
** **	350–500 cells/mm3	1283 (14)
** **	≥ 500 cells/mm3	630 (7)
** **	Not recorded	2269 (19)
**ART Regimen used**	d4T+3TC+NVP	140 (1)
** **	d4T+3TC+EFV	367 (3)
** **	AZT+3TC+NVP	295 (3)
** **	AZT+3TC+EFV	329 (3)
** **	TDF+3TC+NVP	67 (1)
** **	TDF+3TC+EFV	9187 (82)
** **	TDF+FTC+EFV	164 (1)
** **	ABC+3TC+EFV	521 (5)
** **	Other regimen	94 (1)
** **	Not recorded	22 (0)
**Outcomes for those on ART at the end of the study**		11186 (95)
** **	Under Care	8536 (76)
** **	Died	961 (9)
** **	Lost to follow up	1371 (12)
** **	Transferred out	318 (3)

ART, anti-retroviral therapy; HIV, human immunodeficiency virus; MSM, men who have sex with men; SW, sex worker; kg, kilogram; g/dl, gram per deciliter; cells/mm3, cells/cubic milliliter; d4T, stavudine; AZT, zidovudine; ABC, abacavir; TDF, tenofovir; 3TC, lamivudine; FTC, emtricitibine; EFV, efavirenz, NVP, nevaripine.

### Retention in care after ART initiation

Of PLHIV on ART, 79% were retained in care during the study period and the rates (%) of retention after ART initiation were 86 (85–87), 82 (81–83), 80 (79–80), 78 (77–78), and 75 (73–76) at 6, 12, 18, 24 and 36 months respectively.

### Early mortality

Of those initiated on ART, there were 733(6%) early deaths, increasing to 961(9%) over the study period. Being male, aged between 41–50 and ≥51 years, having low body weight at baseline, those not married, habitual alcohol use, WHO stage 3 and 4 disease, being bedridden and low CD4 count (<200 cells/mm3) were independently associated with early death within 6 months after ART initiation. (Table 2)

**Table 2:** Factors associated with early mortality within 6 months after ART initiation among people living with HIV who were enrolled in three hospitals specializing for HIV care in Yangon, Myanmar, 1^st^ June 2013 to 30^th^ June 2016.

Socio-demographic characteristics		n (%)	Incidence Rate* (95% CI)	Unadjusted HR (95% CI)	p-value	Adjusted HR (95% CI)	p-value
**Total**		733 (6)	46 (42–49)				
**Sex**	Female	264 (36)	38 (34–43)	Reference		Reference	
** **	Male	469 (64)	51 (47–56)	**1.32 (1.13–1.54)**	<0.001	**1.84 (1.44–2.35)**	<0.001
**Age at enrollment**	≤25	49 (7)	35 (27–47)	0.82 (0.6–1.12)	0.212	0.79 (0.5–1.25)	0.313
** **	26–30	101 (14)	32 (26–40)	0.81 (0.64–1.02)	0.076	1.06 (0.79–1.43)	0.697
** **	31–40	292 (40)	40 (36–45)	Reference		Reference	
** **	41–50	193 (26)	52 (45–60)	**1.26 (1.05–1.52)**	**0.015**	**1.38 (1.07–1.78)**	**0.013**
	≥51	98 (13)	69 (56–85)	**1.65 (1.31–2.09)**	**<0.001**	**1.68 (1.21–2.34)**	**0.002**
**Baseline Weight in Kg**	<44 Kg	188 (40)	70 (62–80)	**2.88 (2.36–3.51)**	<0.001	**2.06 (1.64–2.59)**	<0.001
** **	44–56 Kg	232 (50)	24 (20–27)	Reference		Reference	
** **	>56 Kg	47 (10)	13 (10–18)	**0.53 (0.38–0.74)**	<0.001	0.82 (0.58–1.16)	0.258
**Year of ART initiation**	2013	111 (15)	34 (28–41)	Reference			
** **	2014	240 (33)	36 (31–41)	**0.76 (0.61–0.96)**	0.021		
** **	2015	266 (36)	54 (47–61)	**0.71 (0.57–0.89)**	0.003		
** **	2016	116 (16)	106 (88–127)	**0.73 (0.56–0.96)**	0.022		
**Marital status**	Single	327 (45)	62 (54–72)	**1.76 (1.47–2.12)**	<0.001	**1.33 (1.04–1.71)**	0.026
** **	Married	197 (27)	34 (31–39)	Reference		Reference	
** **	Separated/Divorced	133 (18)	66 (55–79)	**1.79 (1.45–2.2)**	<0.001	1.32 (1–1.74)	0.053
** **	Widowed	74 (10)	52 (41–65)	**1.63 (1.27–2.1)**	<0.001	1.15 (0.76–1.74)	0.513
**Literacy status**	Illiterate	98 (13)	57 (46–70)	**1.42 (1.14–1.77)**	0.002		
** **	Literate	631 (87)	44 (41–48)	Reference			
**Employment status**	Employed	532 (73)	45 (42–50)	Reference			
** **	Unemployed	194 (27)	45 (39–52)	1.02 (0.86–1.2)	0.843		
**Alcohol consumption**	Habitual**	89 (12)	106 (86–131)	**2.23 (1.77–2.81)**	<0.001		
** **	Social	169 (23)	46 (39–53)	1.02 (0.85–1.23)	0.83		
** **	Never	469 (65)	41 (38–45)	Reference			
**History of previous ART**	No	681 (95)	48 (44–52)	Reference			
** **	Yes	39 (5)	24 (18–34)	**0.5 (0.36–0.7)**	<0.001		
**Baseline Performance**	Normal activity	428 (59)	29 (26–32)	Reference		Reference	
** **	Bedridden less than 50%	64 (9)	296 (229–383)	**7.44 (5.65–9.8)**	<0.001	**3.81 (2.57–5.66)**	<0.001
** **	Bedridden for more than 50%	237 (33)	224 (197–256)	**6.61 (5.62–7.78)**	<0.001	**2.63 (1.64–4.2)**	<0.001
**WHO clinical stage**	Stage 1 + 2	148 (20)	21 (18–25)	Reference		Reference	
** **	Stage 3 + 4	580 (80)	68 (62–74)	**3.15 (2.63–3.78)**	<0.001	1.22 (0.95–1.57)	0.115
**Baseline Hemoglobin level**	<7 g/dl	110 (16)	175 (144–211)	**0.28 (0.22–0.36)**	<0.001	1.25 (0.91–1.73)	0.171
** **	7–11 g/dl	484 (71)	56 (51–62)	Reference		Reference	
** **	>11 g/dl	84 (12)	17 (14–21)	**2.69 (2.18–3.33)**	<0.001	**0.47 (0.34–0.65)**	<0.001
**Baseline CD4 counts**	≤50 cells/mm3	333 (50)	117 (105–131)	**18.67 (7.72–45.18)**	<0.001	**6.83 (2.52–18.57)**	<0.001
** **	51–200 cells/mm3	265 (40)	55 (49–62)	**9.37 (3.87–22.71)**	<0.001	**4.04 (1.49–10.98)**	0.006
** **	201–350 cells/mm3	51 (8)	15 (11–19)	**2.72 (1.08–6.81)**	0.033	2.02 (0.72–5.69)	0.183
** **	350–500 cells/mm3	15 (2)	8 (5–13)	1.26 (0.46–3.47)	0.654	1.08 (0.34–3.44)	0.899
** **	≥ 500 cells/mm3	5 (1)	7 (3–16)	Reference		Reference	
**ART Regimen used**	d4T based Regimen	590 (81)	44 (41–48)	**1.65 (1.24–2.2)**	0.001		
** **	AZT based Regimen	53 (7)	50 (38–66)	**0.61 (0.4–0.93)**	0.021		
** **	ABC Based Regimen	24 (3)	24 (16–36)	**1.98 (1.51–2.61)**	<0.001		
** **	TDF Based Regimen	61 (8)	128 (98–166)	Reference			
** **	Other regimen	4 (1)	27 (9–83)	0.53 (0.17–1.64)	0.271		

*rates were expressed in 1000 person-years of follow-up

**Habitual alcohol consumption means he or she is used to drink regularly.

ART, anti-retroviral therapy; HIV, human immunodeficiency virus; 95% CI, 95% confidence interval; HR, hazard ratio; kg, kilogram; g/dl, gram per deciliter; cells/mm3, cells/cubic milliliter; d4T, stavudine; AZT, zidovudine; ABC, abacavir; TDF, tenofovir.

### Attrition (death and loss to follow up)

**Fig 1:**
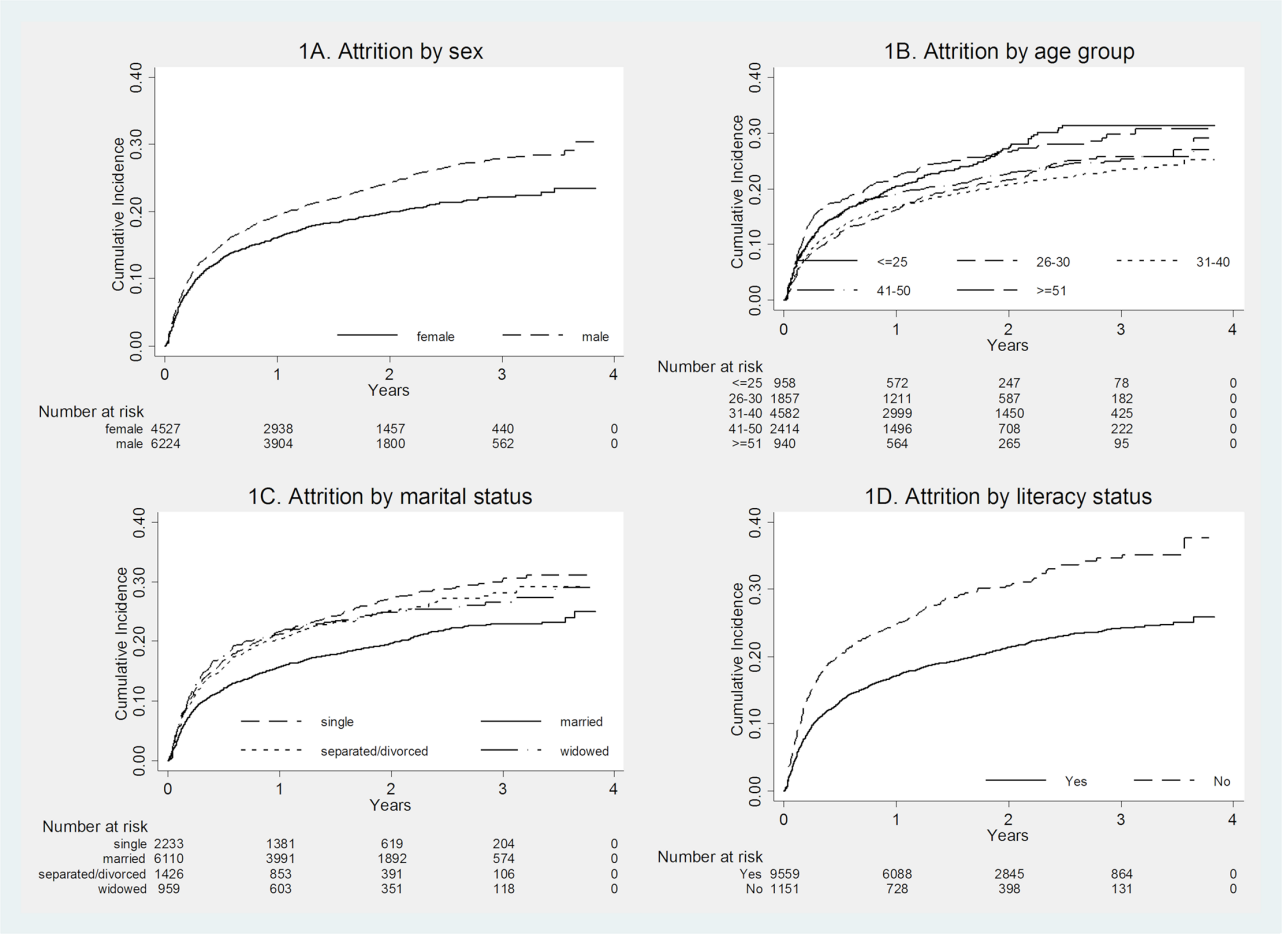
Cumulative incidences of attrition after ART initiation of adult PLHIV in three hospitals specializing for HIV care in Myanmar, 1^st^ June 2013 to 30^th^ June 2016. ART, anti-retroviral therapy; HIV, human immunodeficiency virus; PLHIV, people living with HIV.

**Fig 2:**
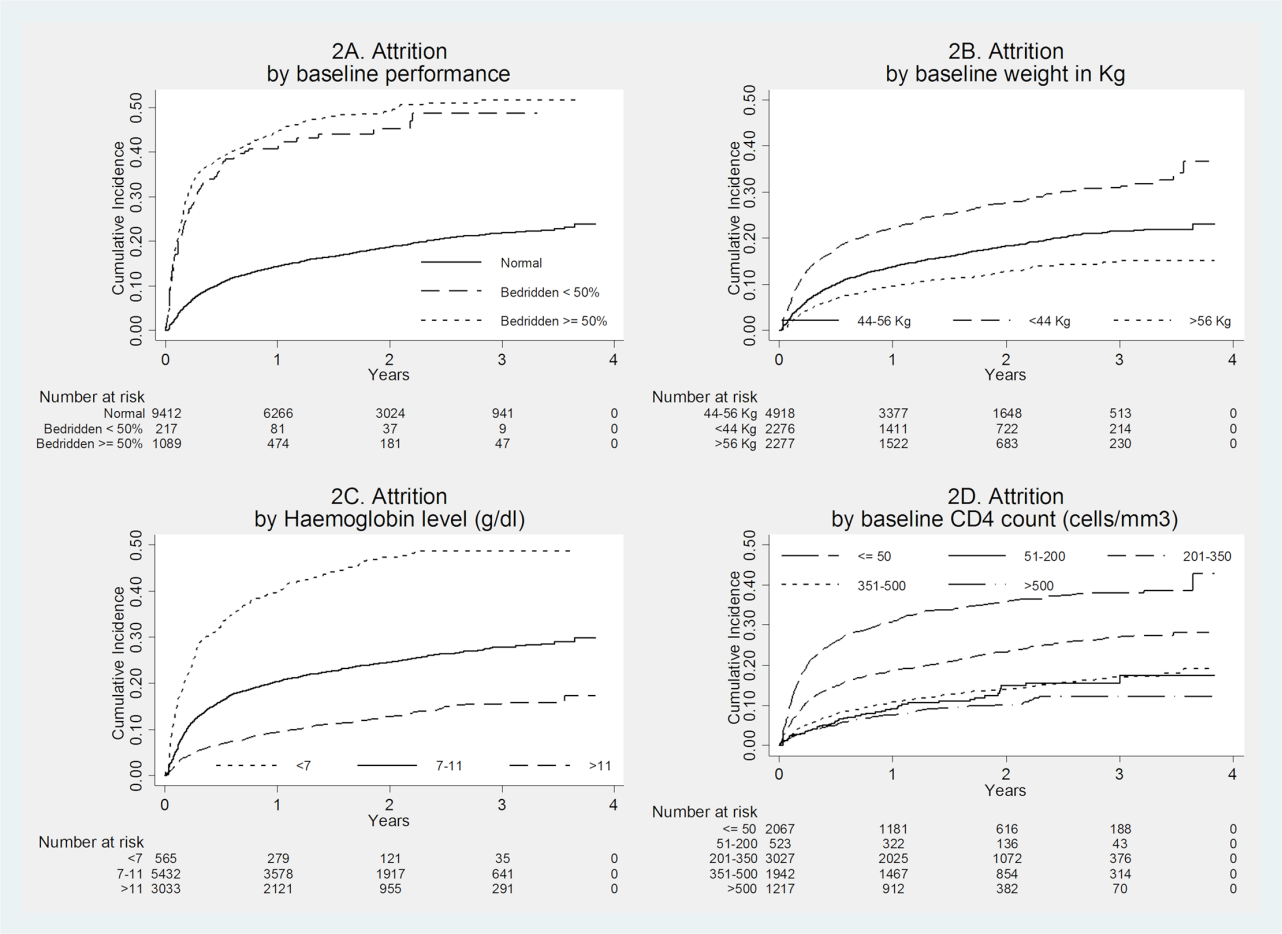
Cumulative incidences of attrition after ART initiation of adult PLHIV in three hospitals specializing for HIV care in Myanmar, 1^st^ June 2013 to 30^th^ June 2016. ART, anti-retroviral therapy; HIV, human immunodeficiency virus; PLHIV, people living with HIV; kg, kilogram; g/dl, gram per deciliter; cells/mm3, cells per cubic milliliter.

Of those initiated on ART, 2332(20%) had undergone attrition (death and loss to follow-up). Overall incidence of attrition was 140 per 1000 person-years. Table 3 shows incidence rate of attrition. Similar factors that were associated with early death were also found to be independently associated with attrition after ART initiation with an addition factor, ABC-based regimen. The unadjusted cumulative incidences of unfavorable outcomes by age, sex, marital status, literacy status, baseline performance scale, weight, haemoglobin level and CD4 count over the period of follow-up are presented in Figs 1 and 2.

**Table 3:** Factors associated with attrition after ART initiation after ART initiation among people living with HIV who were enrolled in three hospitals specializing for HIV care in Yangon, Myanmar, 1^st^ June 2013 to 30^th^ June 2016.

Socio-demographic characteristics		n (%)	Incidence Rate* (95% CI)	Unadjusted HR (95% CI)	p-value	Adjusted HR (95% CI)	p-value
Total		2332 (20)	140 (134–146)				
Sex	Female	876 (38)	121 (113–130)	Reference		Reference	
	Male	1456 (62)	154 (146–163)	**1.25 (1.15–1.36)**	<0.001	**1.59 (1.38–1.84)**	<0.001
Age at enrollment	≤25	245 (11)	175 (154–199)	**1.32 (1.14–1.52)**	<0.001	**1.51 (1.24–1.83)**	<0.001
	26–30	395 (17)	132 (120–147)	1.04 (0.92–1.18)	0.505	1.11 (0.95–1.31)	0.187
	31–40	916 (39)	127 (119–136)	Reference		Reference	
	41–50	533 (23)	146 (133–159)	**1.13 (1.02–1.26)**	0.025	1.12 (0.97–1.3)	0.124
	≥51	243 (10)	176 (155–200)	**1.36 (1.18–1.58)**	<0.001	**1.38 (1.14–1.68)**	0.001
Baseline Weight in Kg	<44 Kg	619 (35)	179 (165–194)	**1.66 (1.49–1.84)**	<0.001	**1.49 (1.31–1.69)**	<0.001
	44–56 Kg	862 (49)	106 (99–113)	Reference		Reference	
	>56 Kg	268 (15)	73 (64–82)	**0.67 (0.58–0.77)**	<0.001	**0.7 (0.59–0.84)**	<0.001
Year of ART initiation	2013	340 (15)	100 (89–111)	Reference			
	2014	843 (36)	122 (114–131)	0.98 (0.86–1.12)	0.775		
	2015	874 (37)	171 (159–182)	1.02 (0.89–1.16)	0.803		
	2016	275 (12)	240 (212–271)	0.96 (0.81–1.13)	0.617		
Marital status	Single	1167 (50)	174 (160–189)	**1.41 (1.27–1.56)**	<0.001	**1.22 (1.06–1.41)**	0.005
	Married	576 (25)	121 (114–128)	Reference		Reference	
	Separated/Divorced	349 (15)	165 (148–184)	**1.31 (1.16–1.48)**	<0.001	**1.26 (1.07–1.48)**	0.005
	Widowed	235 (10)	152 (133–173)	**1.31 (1.14–1.51)**	<0.001	1.22 (1–1.5)	0.052
Literacy status	Illiterate	359 (15)	198 (178–220)	**1.55 (1.38–1.74)**	<0.001	**1.55 (1.33–1.8)**	<0.001
	Literate	1961 (85)	133 (127–139)	Reference		Reference	
Employment status	Employed	1669 (72)	138 (132–145)	Reference			
	Unemployed	650 (28)	145 (134–156)	1.06 (0.97–1.16)	0.209		
Alcohol consumption	Habitual	208 (9)	233 (203–268)	**1.61 (1.39–1.87)**	<0.001	**1.67 (1.37–2.04)**	<0.001
	Social	550 (24)	145 (133–158)	1.05 (0.95–1.16)	0.372	1.05 (0.90–1.21)	0.552
	Never	1549 (67)	131 (125–138)	Reference		Reference	
History of previous ART	no	2095 (91)	142 (136–148)	Reference			
	yes	207 (9)	123 (107–142)	**0.86 (0.75–1.00)**	0.048		
Baseline Performance	Normal activity	1685 (73)	111 (106–116)	Reference		Reference	
	Bedridden less than 50%	99 (4)	450 (367–552)	**3.4 (2.76–4.19)**	<0.001	**2.31 (1.68–3.17)**	<0.001
	Bedridden for more than 50%	532 (23)	463 (425–505)	**3.69 (3.34–4.07)**	<0.001	**2.01 (1.43–2.81)**	<0.001
WHO clinical stage	Stage 1 + 2	720 (31)	95 (89–103)	Reference		** **	
	Stage 3 + 4	1588 (69)	179 (170–188)	**1.85 (1.69–2.02)**	<0.001	** **	
Baseline Hemoglobin level	<7 g/dl	262 (13)	381 (337–431)	**2.24 (1.95–2.56)**	<0.001	**1.30 (1.06–1.59)**	0.012
	7–11 g/dl	1344 (68)	152 (144–160)	Reference		Reference	
	>11 g/dl	375 (19)	74 (67–82)	**0.47 (0.42–0.53)**	<0.001	**0.64 (0.55–0.74)**	<0.001
Baseline CD4 counts	≤50 cells/mm3	742 (39)	251 (233–270)	**3.35 (2.57–4.36)**	<0.001	**1.69 (1.24–2.31)**	0.001
	51–200 cells/mm3	705 (37)	141 (131–152)	**1.97 (1.52–2.57)**	<0.001	1.12 (0.82–1.52)	0.476
	201–350 cells/mm3	281 (15)	77 (68–87)	1.13 (0.86–1.5)	0.377	0.96 (0.70–1.32)	0.805
	350–500 cells/mm3	117 (6)	60 (50–72)	0.80 (0.59–1.10)	0.17	0.82 (0.58–1.16)	0.267
	≥ 500 cells/mm3	66 (3)	78 (61–100)	Reference		Reference	
ART Regimen used	d4T based Regimen	1932 (83)	140 (134–146)	1.05 (0.88–1.26)	0.594	1.01 (0.81–1.25)	0.94
	AZT based Regimen	128 (6)	116 (97–138)	**0.75 (0.61–0.92)**	0.006	0.81 (0.61–1.08)	0.145
	ABC Based Regimen	103 (4)	97 (80–118)	**1.61 (1.35–1.92)**	<0.001	**1.24 (0.97–1.58)**	0.08
	TDF Based Regimen	145 (6)	291 (246–344)	Reference		Reference	
	Other regimen	16 (1)	130 (78–215)	0.86 (0.52–1.42)	0.549	0.79 (0.35–1.77)	0.567

* rates were expressed in 1000 person-years of follow-up.

ART, anti-retroviral therapy; HIV, human immunodeficiency virus; 95% CI, 95% confidence interval; HR, hazard ratio; kg, kilogram; g/dl, gram per deciliter; cells/mm3, cells/cubic milliliter; d4T, stavudine; AZT, zidovudine; ABC, abacavir; TDF, tenofovir.

## Discussion

We report on the largest cohort of PLHIV engaged in hospital-based HIV care in Myanmar. The study has found that 6% of the cohort died at 6 months and 12% were LTFU (20% attrition rate) at the end of the study period. Despite a relatively low early death rate, PLHIV initiated on ART had a very low baseline CD4 indicating delayed access to diagnosis and care.

### Socio-demographic and clinical characteristics

The socio-demographic characteristics of PLHIV were consistent with previous studies and national data, with the notable difference of low percentages of key populations in the cohort.[6] There was a large proportion of missing data on HIV transmission risk factor recorded, largely from Mingalardon specialist hospital. The national survey shows HIV prevalence of 28.5% among people who inject drugs^,^ 14.6% among female sex workers and 11.6% among men who have sex with men.[11] This may have been due to access barriers of these populations to government hospitals or under-reporting of risk factors and behaviors to clinicians, due to fear of discrimination and legal implications from their sexual orientation and drug-use.

A majority of patients in this study had advanced HIV disease with either WHO stage 3 and 4 on presentation, consistent with 2 previous cohort studies in Myanmar conducted since 2006.[5,6] It is concerning that this finding remains consistent despite the scale-up of the HIV testing and treatment services and changes to guidelines for CD4 criteria for ART initiation since the previous studies. This need to be explored further but may be due to delayed presentation to health services due to issues with access and service acceptability or the fact that study sites were tertiary hospitals where the most acute and unwell patients are referred.[12–14]

### Retention in care

The ART retention rate at 12 months of 82% was slightly lower than the national figure in 2010 (87%) and previous reports in Myanmar.[15] Of note, 9% of patients in the cohort had a history of previous ART, indicating ongoing unaddressed adherence needs in the study population. The reasons for lost to care were not explored in this study but may include patient-side and health system factors.[9,16,17] Multiple patient-side factors and socio-economic barriers could play a role and need investigation. As 34% of Yangon’s population are considered mobile or internal migrants[18], this may be important factor to support retention in care.

Possible health system reasons may include limited human resources (clinicians and health care workers) in the government sector for patient support, weak linkages between facility and community-based care, and lack of tools and systems to support monitoring and follow-up of patients in care. The peer volunteers (funded via GFATM) currently provide this function, but the coverage and regularity of their services is not certain, nor sustainable. Currently the country is taking the move to transit from paper-based recording to electronic recording and reporting system using open-source open Medical Record System (openMRS) with added functionality and features such as reminder system and automatic sending of short telephone message.[19–23] In 2013, Stella et al reported that tracing lost-patients on the same day using electronic medical records reduced the LTFU from 11% to 5%. [24]

### Early mortality and associated factors

Overall, 6% of the cohort had an outcome of death at 6 months of follow up after the study period. However, this may be an underestimate as patients who are LTFU may have died. Although overall death rate of 46 per 1000 person-years follow up was higher than other reported cohorts, such as a large study in India (38 per 1000 person-years).[11] We confirmed the well-established strong association between advanced HIV disease (low CD4 count, stage 3 or 4, low weight and bedridden) and death.[25,26] Age 41–50 and ≥50 years was also associated with early mortality. This may have been due to an increased rate of co-morbidities such as smoking and non-communicable diseases in this group.[27–31]

### Attrition and associated factors

Similar predictors for early death were also found to be strongly associated with attrition, which were also reported by previous studies.[5,6] Interestingly, the study showed that being initiated on an ABC-based regimen was strong predictor of attrition, but not mortality, compared with a TDF-based regimen. The reasons for this were not clear, as we did not have data on ART indication. It was unlikely to be a clinically important finding as could have been due to contraindication to first line regimen, clinician choice or drug stock-out; rather than adverse event or ART failure (which would occur after initiation).

### Strengths and limitations

The strengths of this study are that is the largest reported cohort of PLHIV on ART in hospital-based settings from Myanmar, and the first ever from government hospitals specialized in HIV care. Secondly, the data come from government hospitals, thus reflecting successes and challenges for transition of services from projects and NGOs. Thirdly, this study adds to the knowledge based on mortality and retention in care from previous studies conducted from 2006 to 2013. Lastly, we used robust methods of data cleaning and analysis using multivariate survival analysis to understand their associated factors. The Strengthening the Reporting of Observational Studies in Epidemiology (STROBE) guidelines and sound ethics principles for the conduct and reporting of this observational study were used.[32]

This study has several limitations. Firstly, the study population is limited to government hospitals that are located in an urban area, Yangon. The population accessing these services does not represent the majority of PLHIV in Myanmar therefore limiting the generalizability of the findings. Secondly, as the study data was from routine programme records, there were missing data on important variables that could not be assessed such as presence and type of opportunistic infections and HIV viral load. As enrollment date was not consistently recorded, we could not report on time taken to ART initiation. There was a high proportion of missing data in these variables; risk factor for HIV transmission (50%), baseline weight (19%), baseline CD4 count (19%) and baseline haemoglobin (17%). When conducting a missing data analysis, we found that males were more likely to have missing data as compared to females. Patients of age range 31–40 were more likely to have missing CD4 count and hemoglobin results. There may have been underestimation of the risk of weight, CD4 count and haemoglobin on attrition and/or mortality. Missing data is most likely to be related to problems in documentation and patient records, which is currently paper-based. As the country is making an effort to move patients’ records on paper to a web-based electronic medical record system, this missing issue due to data documentation will be largely reduced in the future. As the study data was from routine programme records, there were important variables that we not routinely recorded, such as the presence and type of opportunistic infections and HIV viral load. Finally, as enrollment date was not consistently recorded, we could not report on time taken to ART initiation.

### Implications

The findings are timely and relevant for NAP planning and sustainability, as the Government plans to increase its proportion of financial management of HIV services from 27% in 2015 to more contributions yearly. ART coverage among eligible PLHIV in 2012 was 24% and more than doubled in 2016 (63%). The NAP Myanmar is preparing a transition plan where PLHIV on ART at private sector are to be transferred to public sector over the next 3 years.[2] Although this study reported a low rate of early mortality, the high proportion of PLHIV with advanced HIV needs to be addressed. Concerted effort should be made to get people more engaged in HIV testing, treatment and care. It is encouraging to note that the NAP Myanmar has already determined to adopt the WHO recommendation of a “test and treat” approach (including ART initiation at any CD4 count) which will promote earlier initiation of ART.

To reduce the rates of LTFU, interventions to identify high risk groups and strengthen recording and reporting system of the NAP Myanmar, including individual patient tracking mechanisms together with electronic-based recording and reporting system should be implemented. Qualitative studies to explore the reasons behind LTFU would be important operational research to conduct. Community-based care and home-based care needs to be strengthened with active collaboration and cooperation by PLHIV networks and community-based organizations to create an enabling environment and KP friendly services. The peer counselor project should be evaluated and sustained if successful. In order to monitor the HIV drug resistance and to reach the third 90 target, viral load monitoring system in electronic forms and HIV care cascade should be developed.

## Conclusion

This study demonstrates that successful outcomes are possible for a majority of patients receiving HIV care in government hospitals in Yangon, Myanmar. However, concerted efforts are needed by the NAP and partners to provide quality patient care, improve outcomes and strengthen systems in order to achieve the ambitious plan of zero deaths and 90-90-90 targets by 2020.
